# Malarial Hæmaturia

**Published:** 1886-08-20

**Authors:** Charles C. Thornton

**Affiliations:** Thornton, Holmes county, Miss.


### MALARIAL HÆMATURIA.

BY CHARLES C. THORNTON, OF THORNTON, HOLMES CO., MISS.

I regard this disease as a congestion. We have congestion of the brain, of the stomach, of the bowels, of the lungs, and why not of the kidneys?

Almost all the cases that I have seen have either intermittent fever for some time, and are taken with a severe chill, or are taken from the commencement with a severe chill, and in almost every case, before hemorrhage from the kidneys supervenes, the urine is charged with bile. This is apparent when the urine is made to flow in a thin surface over the bottom of a clean white chamber, porcelain or iron-stone vase. The greenish cast, apparent to the observer, shows a redundancy of bile. Besides this physical change in the condition of the urine, the skin takes on the yellow cast so common in such cases, which only disappears when the bowels act freely; and black, tarry bile passes freely from them, and the skin begins then to clear up, and a return to health is generally the result.

It is this acrid bile passing through the ureters and body of the kidney, the result of a congestion of the liver, or interference with the portal circulation, and the effort of nature to pass this effete matter through the urinary tract, causes the congestion and hemorrhage from the kidney.

Twenty-six years here in a miasmatic region (the heart of the Yazoo Valley), convinces me that my theory has at least the shadow of truth or right, and then when we put the crucial test upon it, the result of my treatment, I think it is proof positive, without entering into any of the details of the symptoms. It is generally those that have had intermittent fevers for sometime and neglected these, that have or are most liable to have malarial hæmaturia, and among the first symptoms are the incessant vomiting of bilious matter and the sudden discoloration of the skin, frequently in six hours after the onset of the attack, and the intense yellow, muddy appearance of the eyes, while the urine is loaded with bile, and hemorrhage soon appears, sometimes almost entirely blood, and in large quantity. As soon as I can get the bowels discharging freely *black* bile, I feel I have reached the point to hope for a favorable termination.

Now, to the main point, the treatment: With a view to get up such an action on the liver and bowels to divert the bile from this unnatural to its proper channel, I give 5 to 10 grains of calomel with 1 grain each of pulverized camphor and ipecac. every three to four hours; I give lime water and fresh milk freely, or lime water alone, to correct or check the constant sick stomach; and when I can get the stomach sufficiently quiet, I give chlorate of potash and fld. ext. ergot freely; that is to a goblet of water I add a tablespoonful chlorate potash, and a tablespoonful fld. ext. ergot, of this I give generally a tablespoonful every two hours; I give it, when the stomach is constantly irritable and there is vomiting; if I cannot get it to stick by the mouth, I use No. 1 rectal capsules, or even the ordinary No. 1 gelatine capsules by the rectum. I generally give three or four of the calomel powders, and wait twelve to twenty-four hours and resort to it again if necessary. There is generally little or no absorption by the stomach, and that is why so much quinine and other medicines are taken, and so little effect. The bowels are usually obstinately constipated; if this is not corrected generally at once, suppression of urine is apt to ensue, and death as a consequence.

Is my treatment rational, empirical, or otherwise? I have the satisfaction of knowing as yet, after twenty-six years experience, I have lost no patients of this disease. My friends may say, Oh, he has never seen the disease, or not in such forms as we see it. Yes, I have seen it in all its phases. When there is a tendency to suppression of urine, I add bicarb. potash to my chlorate and ergot solution. Last year I had three cases in one house on my own place; they passed chambers almost full of blood, and six times during the day, and sometimes oftener; but to use a common term, as soon as the block is "knocked out," they are generally all right. The three cases above are now living monuments of the above practice.

I give these cases my undivided attention. I never want but one of such cases at a time.

Again to the first point, or a probable cause. The first case last year was a little girl, taken with chills, had them sometimes every day, or every other day, when hæmaturia set in. She had two well marked exacerbations, as I term them, sinking spells, each day; generally at 12 o'clock in the day, and 12 o'clock at night, she would get cold, and the heart fag; then I would resort to brandy and milk, keeping up quinine by rectum every two hours; her temperature in axilla 102° to 105°, with quinine, would reduce to 98° each day; but at each paroxysmal time would again rise to 105°, and without quinine, would continue 103° to 105°. After bowels acting freely, black tarry actions, and water perfectly clear, the regular intermittent paroxysm would set in, and controlled by quinine, returned each seventh day for several weeks, and had to continue quinine three times a day all the summer and fall. Another, her brother, had chills; was exposed to a cold rain, had a terrible chill, and hæmaturia set in; same treatment, same success, and same results, and same tendency when well. His father exposed to same cold rain, and worry nursing, and anxiety about his boy, had a hard chill, and the consequences alike, I paid little attention to the bowels, even when running off almost incessantly, while these black tarry actions were passing, as the skin cleared after each action. I never use *opiates*, and warn my friends not to tamper with them. A fatal coma, and often suppression of urine, are too frequently the result of the useless administration of a single dose of opium, as I have seen, under the impression that it would relieve some of the ditressing symptoms. Calomel and quinine are my sheet anchors in this disease, while ergot and chlorate of potash assist to keep up the action of the liver, and lessen the irritation of the stomach, and control measurably the hemorrhage; but I find, as the bile is diverted to its natural channel, the hemorrhage slackens and disappears. I give quinine as anti-ferment, for its anti-zymotic influence, and to keep the temperature within bounds. I trust only to the clinical thermometer, and register temperature every two or three hours, and watch the rise and fall, and combat these as other symptoms, the fall with brandy and milk, the rise with quinine. I give quinine all the time, in fact, and the ergot and chlorate whenever it will stick, and calomel as above, or alone, every three, four or six hours, as required.

I watch my patients, and never allow any solid food until they are out of danger. I rely on a milk diet mostly—it with lime-water is retained best—chicken and beef extracts or essences I also use in moderation, but rely mostly on milk and gum arabic, with ice to allay terrible irritability of the stomach and incessant vomiting.—*Medical and Surgical Reporter.*

